# Writing the Past, Present, and Future: The Impact of Positive Psychology Expressive Writing on Adolescents’ Time Attitudes

**DOI:** 10.3390/bs16010119

**Published:** 2026-01-14

**Authors:** Xiangling Tu, Bo Wu, Xiaobin Ding, Qixuan Huo, Min Chen

**Affiliations:** 1 School of Psychology, Northwest Normal University, Lanzhou 730070, China; 2022104162@nwnu.edu.cn (X.T.); 202511040199@nwnu.edu.cn (Q.H.); 202521040512@nwnu.edu.cn (M.C.); 2 Faculty of Health and Wellness, City University of Macau, Macau, China

**Keywords:** positive psychology expressive writing, adolescents, time attitudes, randomized controlled trial

## Abstract

This study aimed to examine the interventional effects of positive psychology expressive writing (PPEW) on adolescents’ time attitudes and mental health. A total of 285 adolescents from Northwest China (*M* = 14.13, *SD* = 1.075; 53.3% female) were randomly assigned to either a PPEW group (*n* = 148) or a control group (*n* = 137). The PPEW group completed a six-week positive psychology expressive writing intervention, while the control group engaged in neutral writing tasks. All participants were assessed on time attitudes, positive affect, and depressive symptoms before and after the intervention. The results showed that, compared to the control group, the PPEW group scored significantly higher on Past Positive, Present Positive, and Future Positive, and significantly lower on Present Negative at post-test; however, a significant improvement in Past Negative was observed only within the PPEW group itself. Regarding mental health, depressive symptoms were significantly reduced in the PPEW group relative to the control group at post-test, but no significant change was observed in positive affect. In conclusion, positive psychology expressive writing can effectively foster the positive development of time attitudes in adolescents and may serve as a feasible approach to alleviating depressive symptoms.

## 1. Introduction

In recent years, adolescent mental health issues have shown a significant increasing trend (Bommersbach et al., 2023) and have become a public health concern of global importance (Golden et al., 2024; McGorry et al., 2024). Among the various factors associated with adolescent psychological functioning, time attitudes—defined as an individual’s emotional experiences and cognitive evaluations of the past, present, and future (Sommerfeld et al., 2023)—have been identified as a key indicator for assessing and predicting mental health status (Moon et al., 2023; Tejada-Gallardo et al., 2021). Therefore, exploring interventions targeting time attitudes may serve as an important mechanism for improving adverse mental health outcomes in adolescents.

### 1.1. Time Attitude

The adolescent time perspective model posits that time perspective is a multidimensional construct encompassing time attitude, time orientation, time relation, time frequency, and time meaning (Moon et al., 2023). Among these, time attitude serves as its core component, comprehensively reflecting an individual’s affective evaluation across different temporal dimensions, specifically including six subdimensions: Past Positive, Past Negative, Present Positive, Present Negative, Future Positive, and Future Negative. Research indicates that positive attitudes toward the past, present, and future are significantly correlated with higher academic achievement (Andretta & Worrell, 2022), stronger self-efficacy (Worrell et al., 2021), and better mental health outcomes (Sommerfeld et al., 2023). Conversely, negative attitudes toward the past, present, and future are closely associated with higher perceived stress, academic procrastination (J. Wang & Sun, 2023), and risk behaviors such as substance use (Froiland et al., 2021; Finan et al., 2022).

Further research utilizing person-centered analytical methods has revealed distinct profiles of adolescent time attitudes. For example, Tejada-Gallardo et al. (2021) identified four profiles among 317 Spanish adolescents: “Negatives” (11%), “Positives” (45%), “Past Negatives” (17%), and “Present/Future Negatives” (27%). Among them, adolescents with Positives profile scored the highest in well-being and the lowest in psychological distress.

Mental health during adolescence is dynamic (Armitage et al., 2023), and time attitudes are similarly unstable, potentially becoming more negative over time (Zhang et al., 2025), which can further lead to more adverse mental health outcomes (Moon et al., 2023). This may also imply that time attitudes are malleable during adolescence. Indeed, empirical studies have shown that interventions targeting adolescents’ time attitudes are feasible and effective, providing evidence-based support for related practices. For example, Tejada-Gallardo et al. (2022) developed a positive psychology-based intervention program centered on three themes: “expressing gratitude” (past), “character strengths” (present), and “best possible self” (future). The results indicated that adolescents who participated in the intervention were more likely to transition toward a more positive time attitude profile and reported higher levels of well-being.

However, the study validated the intervention’s effectiveness from the perspective of individual heterogeneity and did not systematically examine its differential effects across the six specific dimensions. In fact, the relational patterns between different time attitude dimensions and psychological functioning may vary. For example, past negative attitudes are more strongly associated with internalizing and externalizing problems (Remondi et al., 2026), while future positive attitudes show closer links to well-being and sense of meaning in life (Zong et al., 2022; Newton et al., 2023). Notably, a recent study employing psychodynamic counseling as the intervention strategy found that after completing the intervention, students in the clinical group showed a significant increase in both present positive and future positive, along with a significant reduction in present negative (Sciabica et al., 2025). These findings collectively suggest that comprehensive intervention measures may exert differential effects on distinct dimensions of time attitude. Therefore, it is necessary to further clarify the independent effects of interventions on each of the six specific dimensions.

### 1.2. Positive Psychology Interventions and Expressive Writing

Positive psychology focuses on human psychological strengths, emphasizing the enhancement of social adaptability through fostering positive qualities such as hope and optimism. A variety of evidence-based intervention approaches have been developed in this field, such as expressing gratitude (Choi et al., 2025), acts of kindness (Archer Lee et al., 2024), best possible self (Duffy et al., 2025), self-compassion (Inuwa & Esmaeilzadeh, 2025), and character strengths (Feraco & Casali, 2025). In fact, a growing body of evidence suggests that multi-component positive psychology interventions, which integrate several intervention elements, are more effective than single-component interventions in enhancing subjective well-being, promoting overall mental health, and alleviating emotional distress (Alkal & Çam, 2024; van Agteren et al., 2021). For example, Chilver and Gatt (2022) integrated three interventions—self-compassion, acts of kindness, and positive reminiscence—and confirmed the significant efficacy of the multicomponent program in enhancing well-being and alleviating distress. Consequently, multi-component positive psychology interventions are regarded as an effective strategy for promoting mental health, contributing to the monitoring and optimization of well-being and psychological adaptation in adolescents (Kwok et al., 2022).

Expressive writing, also known as emotional disclosure, is a self-regulation method for psychological states that uses written language as its form of expression (Qiu et al., 2022). In recent years, positive expressive writing, which emphasizes writing about positive experiences, has been shown to effectively enhance well-being and psychological resilience, particularly among individuals who have experienced stress or adversity (Saldanha & Barclay, 2021; Shang et al., 2021). It has also demonstrated positive intervention effects in adolescent populations (Jones & Destin, 2021).

### 1.3. The Present Study

Based on the analysis above, this study targets time attitude as the intervention focus (Tejada-Gallardo et al., 2022) and integrates positive psychology interventions with expressive writing to develop an intervention program termed positive psychology expressive writing (PPEW). The aim is to explore the effects of PPEW on the six dimensions of time attitudes among Chinese adolescents: Past Positive, Past Negative, Present Positive, Present Negative, Future Positive, and Future Negative.

Furthermore, according to the dual-factor model of mental health (Magalhães, 2024), complete mental health should encompass both low levels of psychopathology and high levels of well-being. This model has received substantial empirical support in adolescent populations (Jefferies et al., 2023). Given the close association between time attitudes and mental health, as well as the documented role of positive writing in promoting mental health, this study includes mental health as a secondary outcome and evaluates intervention effectiveness using both positive affect (as an indicator of well-being) and depressive symptoms (as an indicator of psychopathology).

Based on the above rationale, the following hypotheses are proposed:

**Hypothesis 1:**  *Compared to the control group, adolescents in the PPEW group will show higher scores on positive time attitudes (Past Positive, Present Positive, and Future Positive) and lower scores on negative time attitudes (Past Negative, Present Negative, and Future Negative) after the intervention.*

**Hypothesis 2:**  *Adolescents in the PPEW group will report higher scores on positive affect and lower scores on depressive symptoms after the intervention.*

## 2. Methods

### 2.1. Participants

**Figure 1:**
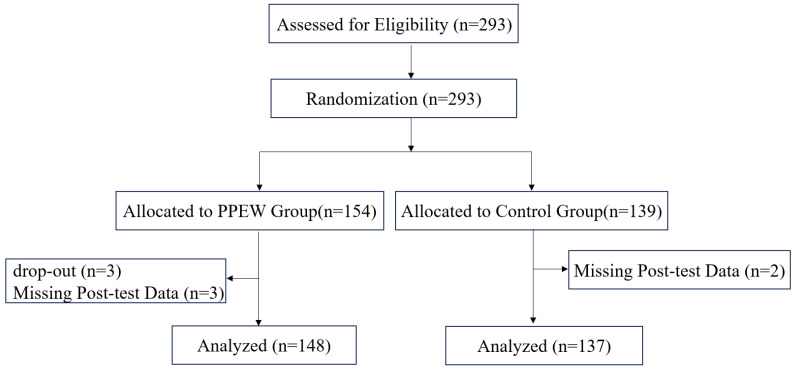
CONSORT (Consolidated Standards of Reporting Trials) flow diagram.

A total of 293 adolescents were assessed for eligibility and enrolled in the study. Participants were randomly assigned to either the PPEW intervention group (*n* = 154) or the control group (*n* = 139). During the intervention phase, three participants in the PPEW group withdrew, and post-intervention data were missing for three additional participants in the PPEW group and two in the control group. Consequently, the final per-protocol analysis included 285 participants (PPEW group: *n* = 148; control group: *n* = 137). Participant flow throughout the trial is detailed in the Consolidated Standards of Reporting Trials (CONSORT) diagram (Figure 1).

The final sample (*n* = 285) had a mean age of 14.13 years (*SD* = 1.075), consisting of 133 males (46.7%) and 152 females (53.3%). The PPEW group comprised 148 participants (*M* = 14.07, *SD* = 1.063), including 65 males, 83 females, with 93 in Grade 7 and 55 in Grade 10. The control group included 137 participants (*M* = 14.19, *SD* = 1.088), with 68 males, 69 females, 80 in Grade 7, and 57 in Grade 10. Independent samples *t*-test and chi-square tests indicated no statistically significant differences between the groups in age (*t* = 0.91, *p* = 0.366), gender [*χ*^2^(1) = 0.93, *p* = 0.334], or grade level [χ^2^(1) = 0.59, *p* = 0.443], confirming successful randomization and baseline comparability.

### 2.2. Research Instruments

#### 2.2.1. Adolescent Time Attitude Scale (ATAS)

Time attitude was assessed using the Chinese version of the Adolescent Time Attitude Scale (Li et al., 2021). The instrument comprises 30 items (e.g., “I look forward to my future.”) that measure six dimensions: Past Positive, Past Negative, Present Positive, Present Negative, Future Positive, and Future Negative, with five items per dimension. Responses were recorded on a 5-point Likert scale ranging from 1 (completely disagree) to 5 (completely agree). Higher scores on the Past Positive, Present Positive, and Future Positive subscales indicate more positive time attitudes, while higher scores on the Past Negative, Present Negative, and Future Negative subscales reflect more negative time attitudes. In this study, the Cronbach’s α values for the six dimensions were as follows: at pretest: 0.804, 0.817, 0.839, 0.817, 0.867, 0.685; at post-test: 0.820, 0.864, 0.831, 0.820, 0.865, 0.760. Given the borderline reliability of the Future Negative subscale at pretest (α = 0.685), we further computed McDonald’s ω as an indicator of composite reliability. The McDonald’s ω values at pretest were 0.805, 0.817, 0.837, 0.816, 0.868, 0.686, and at post-test were 0.816, 0.865, 0.831, 0.821, 0.866, 0.773. These results indicate acceptable reliability across all subscales, consistent with prior research noting relatively lower internal consistency for the Future Negative dimension compared to others (e.g., Przepiórka et al., 2022).

#### 2.2.2. Positive and Negative Affect Scale for Children (PANAS-C)

Positive affect was assessed using the positive affect subscale of the Chinese version of the Positive and Negative Affect Scale for Children (Pan et al., 2025). This subscale consists of 15 items, each presenting a single positive emotion word (e.g., “Happy”). Responses were recorded on a 5-point Likert scale ranging from 1 (“very slight or none at all “) to 5 (“extremely strong “). Higher scores indicate higher levels of positive affect. In this study, the Cronbach’s α for the pretest and post-test were 0.926 and 0.930, respectively.

#### 2.2.3. Depression-Anxiety-Stress Scales-21 (DASS-21)

Depressive symptoms were assessed using the depression symptoms subscale of the Chinese version of the Depression-Anxiety-Stress Scales-21 (DASS-21, Xie et al., 2022). This subscale comprises 7 items (e.g., “I felt that life was meaningless.”). Responses are rated on a 4-point scale ranging from 0 (“Did not apply to me”) to 3 (“Applied to me most”).

For analysis, the item mean score was used, yielding a possible range from 0 to 3, with higher scores indicating more severe depressive symptoms. In the present study, the Cronbach’s α for this subscale at pretest and post-test were 0.794 and 0.836, respectively.

### 2.3. Procedure

This study recruited participants through school-wide announcements, and both adolescent assent and parental written informed consent were obtained. All eligible participants were enrolled. Randomization was conducted by an independent research assistant not involved in subsequent data collection or intervention delivery, using a computer-generated random number sequence to assign participants to either the PPEW intervention group or the control group. The allocation scheme was concealed in sealed envelopes after generation. Baseline assessments were completed before the intervention commenced, and post-intervention evaluations were conducted immediately after the program ended. Both assessment (questionnaire administration) and intervention delivery were carried out by two uniformly trained master’s students in psychology, with outcome assessors remaining blinded to participants’ group assignments throughout the study.

During evening self-study sessions, participants in the PPEW group and the control group completed the writing tasks in their respective assigned classrooms. Each session began after a research assistant read out the standardized instructions, and was conducted under full supervision to ensure compliance, address any questions, and maintain the independence of task execution. To prevent contamination, participants were instructed not to discuss the content of the tasks. There was no word limit for the writing, and free expression was encouraged. Attendance was recorded via on-site sign-in, and only data from participants who completed all six sessions were included in the final analysis.

### 2.4. Writing Tasks

The PPEW group received a six-week positive psychology expressive writing intervention, administered once per week with each session lasting approximately 20 min (Nie et al., 2023). The intervention content was structured around three time-related themes: expressing gratitude, character strengths, and best possible self (Tejada-Gallardo et al., 2022), with each theme addressed in two separate writing sessions. The control group, within the same timeframe, completed writing tasks focused on describing neutral life details.

Expressing ratitude: During the first and second intervention sessions, participants were instructed to write about an experience in which they expressed gratitude toward someone else. They were asked to specifically describe the recipient’s actions, the reasons for their gratitude, and how those actions impacted their lives (Choi et al., 2025). Participants could choose to write about different recipients in each session or deepen their expression toward the same individual.

Character Strengths: In the third and fourth sessions, participants identified their signature strengths and documented specific instances of applying these strengths in daily life. They were also encouraged to experiment with new and varied ways of utilizing these strengths (Feraco & Casali, 2025). Participants could either focus on different strengths in each session or continue exploring the same strength in greater depth.

Best Possible Self: During the fifth and sixth sessions, participants engaged in “best possible self” writing. They were guided to imagine and vividly describe their optimal state in multiple core life domains—such as romantic relationships, career development, physical and mental health, and social life—ten years into the future (Bartha et al., 2025). Participants could choose to concentrate on a single domain or incorporate multiple areas in their narrative.

Life Details: The control group’s writing tasks were adapted from neutral-themed designs used in previous studies (Tornquist et al., 2023). Topics included yesterday’s activities, today’s schedule, or plans for next week. Participants were instructed to objectively describe relevant facts without delving into personal thoughts or emotions.

It is worth noting that the neutral writing task assigned to the control group may itself have a small positive psychological effect (Gerger et al., 2022). To attribute the between-group differences to the specific components of the intervention, this study employed generic titles, neutral instructions, and assessor blinding to control for expectation effects, thereby supporting the conclusion that the observed effects are primarily attributable to the positive psychology exercises.

### 2.5. Data Analysis

Data processing and statistical analysis in this study were conducted using SPSS 25.0. The sample size was determined through a priori power analysis with G*Power 3.1.9.7, with the significance level set at α = 0.05, effect size f^2^ = 0.25, and statistical power (1 – β) = 0.95, resulting in a minimum required sample size of 54 participants.

A 2 (group: intervention/control) × 2 (time: pretest/post-test) repeated measures analysis of variance (ANOVA) was performed, with the dependent variables consisting of six dimensions of time attitudes, positive affect, and depressive symptoms, totaling eight indicators. To control for inflation of Type I error due to multiple comparisons, the Bonferroni correction was applied. The family-wise error rate was set at α = 0.05, resulting in an adjusted significance level of α = 0.00625 for each interaction.

## 3. Results

This study examined the effects of PPEW on the six dimensions of adolescents’ time attitudes and included mental health as a secondary outcome. Since the interaction effect involving future negative attitudes and positive affect did not survive the correction (*p* > 0.00625), it will not be included in the analysis below. The Results section will report the remaining six indicators (five dimensions of time attitudes and depressive symptoms) that passed the correction threshold. Table 1 presents the descriptive statistics for each group at different time points, as well as effect sizes and significance levels for between-group comparisons at post-test. Table 2 displays the within-group comparison results between pretest and post-test for each group.

Concerning Past Positive, the PPEW group improved, whereas the control group declined, with a significant between-group difference observed at post-test. Specifically, the main effect of time was not significant (*F*(1, 283) = 0.417, *p* = 0.519, $\eta_{p}^{2}$ = 0.001), the main effect of group was not significant (*F*(1, 283) = 1.708, *p* = 0.192, $\eta_{p}^{2}$ = 0.006), but the time × group interaction was significant (*F*(1, 283) = 29.122, *p* < 0.001, $\eta_{p}^{2}$ = 0.093). Simple effects analysis revealed no significant difference between groups at pretest (*p* > 0.05). At post-test, the PPEW group scored significantly higher than the control group at post-test (*p* < 0.01). Within-group comparisons showed that the PPEW group’s post-test scores were significantly higher than their pretest scores (*p* < 0.001), whereas the control group’s post-test scores were significantly lower than their pretest scores (*p* < 0.01).

Concerning Past Negative, the PPEW group declined while the control group remained stable, with a non-significant between-group difference observed at post-test. The main effect of time was marginally significant (*F*(1, 283) = 3.702, *p* = 0.055, $\eta_{p}^{2}$ = 0.013), the main effect of group was not significant (*F*(1, 283) = 0.471, *p* = 0.493, $\eta_{p}^{2}$ = 0.002), but the time × group interaction was significant (*F*(1, 283) = 10.409, *p* = 0.001, $\eta_{p}^{2}$ = 0.035). Simple effects analysis showed no significant difference between groups at pretest (*p* > 0.05). At post-test, the PPEW group scored lower than the control group, though this difference was not statistically significant (*p* > 0.05). Within-group comparisons revealed that the PPEW group’s post-test scores were significantly lower than their pretest scores (*p* < 0.001), while no significant difference was found between pretest and post-test scores in the control group (*p* > 0.05).

Concerning Present Positive, the PPEW group improved whereas the control group declined, with a non-significant between-group difference observed at post-test. Specifically, the main effect of time was not significant (*F*(1, 283) = 1.033, *p* = 0.310, $\eta_{p}^{2}$ = 0.004), the main effect of group was not significant (*F*(1, 283) = 0.510, *p* = 0.476, $\eta_{p}^{2}$ = 0.002), but the time × group interaction was significant (*F*(1, 283) = 15.716, *p* < 0.001, $\eta_{p}^{2}$ = 0.053). Simple effects analysis indicated no significant difference between groups at pretest (*p* > 0.05). At post-test, the PPEW group scored significantly higher than the control group (*p* < 0.05). Within-group comparisons showed that the PPEW group’s post-test scores were significantly higher than their pretest scores (*p* < 0.05), whereas the control group’s post-test scores were significantly lower than their pretest scores (*p* < 0.01).

Concerning Present Negative, the PPEW group declined, whereas the control group improved, with a significant between-group difference observed at post-test. Specifically, the main effect of time was not significant (*F*(1, 283) = 0.003, *p* = 0.959, $\eta_{p}^{2}$ = 0.000), the main effect of group was not significant (*F*(1, 283) = 1.103, *p* = 0.294, $\eta_{p}^{2}$ = 0.004), but the time × group interaction was significant (*F*(1, 283) = 16.085, *p* < 0.001, $\eta_{p}^{2}$ = 0.054). Simple effects analysis revealed no significant difference between groups at pretest (*p* > 0.05). At post-test, the PPEW group scored significantly lower than the control group (*p* < 0.05). Within-group comparisons demonstrated that the PPEW group’s post-test scores were significantly lower than their pretest scores (*p* < 0.01), while the control group’s post-test scores were significantly higher than their pretest scores (*p* < 0.01).

Concerning Future Positive, the PPEW group improved whereas the control group declined, with a significant between-group difference observed at post-test. Specifically, the main effect of time was not significant (*F*(1, 283) = 1.461, *p* = 0.228, $\eta_{p}^{2}$ = 0.005), the main effect of group was significant (*F*(1, 283) = 6.098, *p* = 0.014, $\eta_{p}^{2}$ = 0.021), the time × group interaction was significant (*F*(1, 283) = 28.429, *p* < 0.001, $\eta_{p}^{2}$ = 0.091). Simple effects analysis showed no significant difference between groups at pretest (*p* > 0.05). At post-test, the PPEW group scored significantly higher than the control group (*p* < 0.001). Within-group comparisons revealed that the PPEW group’s post-test scores were significantly higher than their pretest scores (*p* < 0.01), whereas the control group’s post-test scores were significantly lower than their pretest scores (*p* < 0.001).

Concerning depressive symptoms, the PPEW group declined, whereas the control group improved, with a significant between-group difference observed at post-test. Specifically, the main effect of time was not significant (*F*(1, 283) = 0.355, *p* = 0.552, $\eta_{p}^{2}$ = 0.001), the main effect of group was not significant (*F*(1, 283) = 2.036, *p* = 0.155, $\eta_{p}^{2}$ = 0.007), but the time × group interaction was significant (*F*(1, 283) = 14.464, *p* < 0.001, $\eta_{p}^{2}$ = 0.049). Simple effects analysis indicated no significant difference between groups at pretest (*p* > 0.05). At post-test, the PPEW group scored significantly lower than the control group (*p* < 0.01). Within-group comparisons demonstrated that the PPEW group’s post-test scores were significantly lower than their pretest scores (*p* < 0.05), whereas the control group’s post-test scores were significantly higher than their pretest scores (*p* < 0.01).

## 4. Discussion

This study combined positive psychology with expressive writing to design a writing intervention program. The program focuses on three core themes: expressing gratitude (past), character strengths (present), and best possible self (future). It aims to systematically examine its effects on adolescents’ multidimensional time attitudes and mental health.

The study found that PPEW can effectively improve adolescents’ attitudes toward the past and present, which aligns with existing research findings (Tejada-Gallardo et al., 2022). Specifically, the intervention group scored significantly higher than the control group on all three positive time attitude dimensions and significantly lower on the past negative and present negative dimensions. However, no significant group difference was observed in the future negative dimension. This is consistent with the results of a recent psychodynamic counseling intervention study, which similarly found that the intervention did not significantly alter participants’ future negative time attitudes (Sciabica et al., 2025). This may be because future negative attitudes are linked to more stable cognitive-affective structures, such as anticipatory anxiety and intolerance of uncertainty (Regnoli et al., 2024). While short-term positive writing can promote future-oriented positive imagery, it may not substantially modify negative future expectations rooted in long-term experiences or cognitive schemas. Indeed, research indicates that longer-term, sustained psychological interventions are more effective in enhancing well-being (Linford & Warren, 2025). Future research could incorporate more targeted modules, such as cognitive bias modification, anxiety regulation, or future scenario simulation, to enhance the intervention’s effectiveness in reconstructing future negative attitudes (Sicouri et al., 2024).

In terms of mental health, PPEW significantly reduced depressive symptoms among adolescents, which aligns with findings from previous research (Shang et al., 2021). It is noteworthy that the control group showed an increase in depressive levels during the intervention period, consistent with the rising trend in the detection rate of adolescent depression in China (M. Wang, 2021). This highlights the practical urgency of implementing preventive psychological interventions. However, the intervention did not significantly enhance positive affect. This outcome supports the perspective of the dual-factor model of mental health, which posits that psychopathology and positive well-being are relatively independent dimensions with distinct underlying mechanisms (Magalhães, 2024). PPEW primarily improved time attitudes and alleviated depression through cognitive restructuring, rather than directly activating the positive emotional system. Relevant research also indicates that expressive writing can alleviate perceived stress and depressive symptoms (Bechard et al., 2021) but has limited effects on enhancing psychological well-being (Sari & Saleh, 2021). Furthermore, the frequency of the intervention may influence its effectiveness. Interventions of shorter writing intervals (e.g., 1–3 days) often yield more stable effects (Guo, 2023), as improvements in positive affect may require more sustained and intensive accumulation of positive experiences.

It is noteworthy that the intervention primarily yielded effect sizes in the small-to-moderate range (e.g., between-group Cohen’s *d* at post-test ranged from 0.219 to 0.519). Effect sizes of this magnitude are typical and practically meaningful within the context of brief, universal, school-based mental health programs aimed at primary prevention. This finding aligns with existing research, indicating that expressive writing had an overall small but significant effect (Hedges’ g = −0.12, 95% CI [−0.21, −0.04]) on reducing symptoms of depression, anxiety, and stress (Guo, 2023). Given the low cost, high accessibility, and ease of implementation of the intervention, even small positive shifts in attitudes and symptoms at the population level may translate into considerable public health benefits.

In summary, this study innovatively integrated the core principles of positive psychology with the operational format of expressive writing to develop and validate a comprehensive intervention program (PPEW). While the independent effectiveness of both positive psychology interventions and expressive writing in adolescent populations has been empirically supported by multiple studies (Alkal & Çam, 2024; Jones & Destin, 2021), existing research has rarely systematically combined these two approaches or designed structured, theme-based interventions based on a comprehensive temporal framework of past, present, and future. This study explicitly targeted adolescents’ multidimensional time attitudes. The results not only revealed the differential effects of PPEW on time attitudes across different dimensions and mental health indicators but also demonstrated that this low-cost, scalable, and self-administered intervention possesses good feasibility and practical value within a school-based, non-clinical adolescent population, offering a novel practical pathway for preventive mental health interventions for adolescents.

This study has the following limitations. First, the sample was drawn from only a few schools in a single region, and data on key contextual factors such as urban-rural setting, school type, and socioeconomic status were lacking. This constrains the external validity and generalizability of the findings. Second, measurement relied entirely on student self-reports, with no multi-source data or behavioral indicators. Third, the study design did not include long-term follow-up or an intervention fidelity check. Furthermore, conducting the intervention in a school setting may have been subject to expectancy effects or the Hawthorne effect. Fourth, the control group completed a neutral writing task rather than serving as a pure no-intervention baseline. Therefore, the extent to which between-group differences can be attributed to the positive components of PPEW should be interpreted cautiously, as some research suggests that neutral writing itself may exert a modest positive influence on psychological state (Gerger et al., 2022).

Future research should aim to enhance the representativeness of samples, incorporate multi-informant data (e.g., teacher or parent ratings) and behavioral indicators, conduct long-term follow-ups and mechanism testing, and consider extending the PPEW intervention period (e.g., to 9–12 weeks) to further validate and strengthen its effectiveness.

## 5. Conclusions

This study examined the effects of a positive psychology expressive writing intervention on adolescents’ time attitudes and mental health. The results demonstrate that the PPEW enhanced positive attitudes toward the past, present, and future, and reduced negative time attitudes toward the past and present. Regarding mental health, the intervention alleviated depressive symptoms, though it did not enhance positive affect. These findings provide empirical support for designing psychological interventions for adolescents from a temporal–cognitive perspective and offer a practical reference for implementing preventive mental health promotion in group settings such as schools.

## Figures and Tables

**Table 1:** Descriptive statistics and between-group comparisons (*M* ± *SD*).

Variable	Time	Control Group	PPEW Group	*p*	*MD* [*CI* 95%]	*Cohen’s d*
Past Positive	Pretest	3.672 ± 0.781	3.601 ± 0.725	0.432	−0.070 [−0.246, 0.105]	−0.094
	Post-test	3.515 ± 0.773	3.800 ± 0.706	0.001	0.285 [0.112, 0.457]	0.385
Past Negative	Pretest	2.349 ± 0.831	2.405 ± 0.839	0.569	0.057 [−0.138, 0.251]	0.067
	Post-test	2.397 ± 0.855	2.215 ± 0.809	0.066	−0.182 [−0.376, 0.012]	−0.219
Present Positive	Pretest	3.448 ± 0.791	3.366 ± 0.785	0.381	−0.082 [−0.266, 0.102]	−0.104
	Post-test	3.270 ± 0.713	3.472 ± 0.778	0.024	0.202 [0.027, 0.376]	0.271
Present Negative	Pretest	2.501 ± 0.757	2.546 ± 0.811	0.628	0.045 [−0.138, 0.229]	0.057
	Post-test	2.638 ± 0.800	2.412 ± 0.746	0.014	−0.226 [−0.406, −0.045]	−0.292
Future Positive	Pretest	3.689 ± 0.791	3.703 ± 0.755	0.882	0.014 [−0.167, 0.194]	0.018
	Post-test	3.451 ± 0.821	3.853 ± 0.726	<0.001	0.402 [0.221, 0.582]	0.519
Depressive Symptom	Pretest	0.604 ± 0.541	0.616 ± 0.578	0.856	0.012 [−0.119, 0.143]	0.021
	Post-test	0.722 ± 0.661	0.530 ± 0.522	0.007	−0.192 [−0.330, −0.053]	−0.322

**Note:** MD refers to mean difference, and Cohen’s d refers to the standardized mean difference between the PPEW group and the control group.

**Table 2:** Descriptive statistics and within-group pre–post-test comparison (*M* ± *SD*).

Variable	Group	Pretest	Post-Test	*p*	*MD* [*CI* 95%]	*Cohen’s d*
Past Positive	Control Group	3.672 ± 0.781	3.515 ± 0.773	0.001	−0.156 [−0.249, −0.063]	−0.202
	PPEW Group	3.601 ± 0.725	3.800 ± 0.706	<0.001	0.199 [0.109, 0.288]	0.278
Past Negative	Control Group	2.349 ± 0.831	2.397 ± 0.855	0.367	0.048 [−0.057, 0.153]	0.057
	PPEW Group	2.405 ± 0.839	2.215 ± 0.809	<0.001	−0.191 [−0.292, −0.090]	−0.231
Present Positive	Control Group	3.448 ± 0.791	3.270 ± 0.713	0.001	−0.178 [−0.280, −0.077]	−0.236
	PPEW Group	3.366 ± 0.785	3.472 ± 0.778	0.034	0.105 [0.008, 0.203]	0.136
Present Negative	Control Group	2.501 ± 0.757	2.638 ± 0.800	0.005	0.137 [0.041, 0.233]	0.176
	PPEW Group	2.546 ± 0.811	2.412 ± 0.746	0.005	−0.134 [−0.226, −0.042]	−0.172
Future Positive	Control Group	3.689 ± 0.791	3.451 ± 0.821	<0.001	−0.238 [−0.341, −0.135]	−0.295
	PPEW Group	3.703 ± 0.755	3.853 ± 0.726	0.003	0.150 [0.051, 0.249]	0.202
Depressive Symptom	Control Group	0.604 ± 0.541	0.722 ± 0.661	0.002	0.118 [0.042, 0.194]	0.195
	PPEW Group	0.616 ± 0.578	0.530 ± 0.522	0.021	−0.086 [−0.159, −0.013]	−0.156

**Note:** MD refers to mean difference, and Cohen’s d refers to the standardized mean difference between pretest and post-test.

## Data Availability

The data presented in this study are available on request from the corresponding author. The data are not publicly available due to privacy concerns.
